# Color Doppler Ultrasonography for the Evaluation of Subclavian Artery Stenosis

**DOI:** 10.3389/fneur.2022.804039

**Published:** 2022-02-17

**Authors:** Jie Zhang, Lijuan Wang, Ying Chen, Sibo Wang, Yingqi Xing, Li Cui

**Affiliations:** ^1^Neuroscience Center, Department of Neurology, The First Hospital of Jilin University, Jilin University, Changchun, China; ^2^Department of Vascular Ultrasonography, Xuanwu Hospital, Capital Medical University, Beijing, China

**Keywords:** color Doppler ultrasonography, subclavian artery stenosis, subclavian steal syndrome, vertebrobasilar insufficiency, digital subtraction angiography

## Abstract

**Background:**

It is of great significance to evaluate symptomatic subclavian artery (SA) stenosis by color Doppler ultrasonography. More than 50% SA stenosis may induce symptoms. Currently, there is a paucity of published literature and lack of practitioner consensus for how ultrasonic findings should be interpreted in patients with SA stenosis.

**Objective:**

The study aimed to prospectively evaluate SA stenosis using color Doppler ultrasonography, with digital subtraction angiography as a reference. Moreover, we aimed to determine the optimal thresholds to predict SA stenosis (≥50%).

**Methods:**

A total of 423 SAs from 234 patients with normal or stenotic lumen were enrolled. The peak systolic velocity (PSV) and acceleration time at the stenotic and distal segments of the SA, peak reversed velocity of the vertebral artery, and waveforms of the stenotic SA, distal SA, and vertebral artery were recorded. The ratios of stenotic PSV to distal PSV (PSVr) and distal AT to stenotic AT were also calculated. The optimal cutoff values were determined using receiver operating characteristic analysis.

**Results:**

All ultrasonic parameters were significantly correlated with the degree of SA stenosis, whereas PSV (*r* = 0.624, *P* < 0.001), PSVr (*r* = 0.654, *P* < 0.001) and VA waveform change (*r* = 0.631, *P* < 0.001) had the strongest correlation with SA stenosis. The optimal cutoff values were as follows: PSV ≥ 230 cm/s and PSVr ≥ 2.2 to predict ≥ 50% stenosis, and PSV ≥ 340 cm/s and PSVr ≥ 3.0 to predict ≥ 70% stenosis.

**Conclusions:**

Symptomatic patients with the ultrasonic parameters of PSV ≥ 230 cm/s and PSVr ≥ 2.2 need to be considered for further verification by computed tomographic angiography or magnetic resonance angiography, or by digital subtraction angiography with a view to percutaneous transluminal angioplasty/stent implantation in the same session. The recommended graded cutoff values can help in long-term management of patients with SA stenosis.

## Introduction

The prevalence of subclavian artery (SA) disease is ~2% in the general population, 42% in patients with documented peripheral artery disease, and 5% in patients referred for coronary artery bypass grafting (CABG). In patients with SA disease, half have concomitant coronary artery disease and one-third have carotid and/or vertebral artery (VA) disease (1–3). SA stenosis is a marker of atherosclerotic disease and increased risk of cardiovascular, cerebrovascular, and peripheral vascular events (3–8). In symptomatic patients with SA stenosis, surgical or interventional treatment should be considered (9, 10). Indications include upper extremity claudication, vertebrobasilar insufficiency, symptoms of myocardial ischemia in patients who underwent CABG utilizing the internal mammary artery, and lower extremity claudication in patients who underwent axillo-femoral bypass. Treatment should also be considered to increase blood flow before surgical procedures, such as CABG or the creation of dialysis arteriovenous fistula (1, 11, 12). Thus, accurate evaluation of SA stenosis is of great importance, especially symptomatic SA stenosis.

There are several non-invasive methods for evaluating SA stenosis. Measuring the blood pressure in both arms is a convenient and effective method of assessing SA stenosis. A systolic cuff pressure difference of >10 mmHg is considered significant. However, there is a high false positive rate in patients with hypertension (13). And the accuracy and precision of blood pressure measurement varies across different measurement sites (14). Radial pulse palpation is another useful clinical examination. But it depends on changes in blood pressure and its accuracy is questionable (15, 16). The current guidelines rate color Doppler ultrasonography (CDU) as appropriate for the evaluation of a patient with suspected subclavian occlusive disease (17). Ultrasonic assessment of stenotic SA enables the detection of high-velocity flow and monophasic post-stenotic flow in SA, and reversed flow in ipsilateral VA (10). To date, there is a lack of practitioner consensus for ultrasonic evaluation of symptomatic SA stenosis and how ultrasonic findings should be interpreted in patients with SA stenosis. Two previous studies only evaluated ≥70% stenosis of SA by CDU (18, 19). However, ≥70% stenosis of SA is not equivalent to symptomatic stenosis. Even 50–69% SA stenosis can induce symptoms, especially when myocardial or upper extremity flow demand increases (4, 20). It is necessary to establish ultrasonic criteria to predict symptomatic SA stenosis.

Therefore, the aim of this study was to prospectively evaluate SA stenosis using CDU, with digital subtraction angiography (DSA) as the reference. We aimed to validate the diagnostic efficacy of CDU and assess the optimal thresholds for ≥50% SA stenosis.

## Materials and Methods

### Population

**Figure 1:**
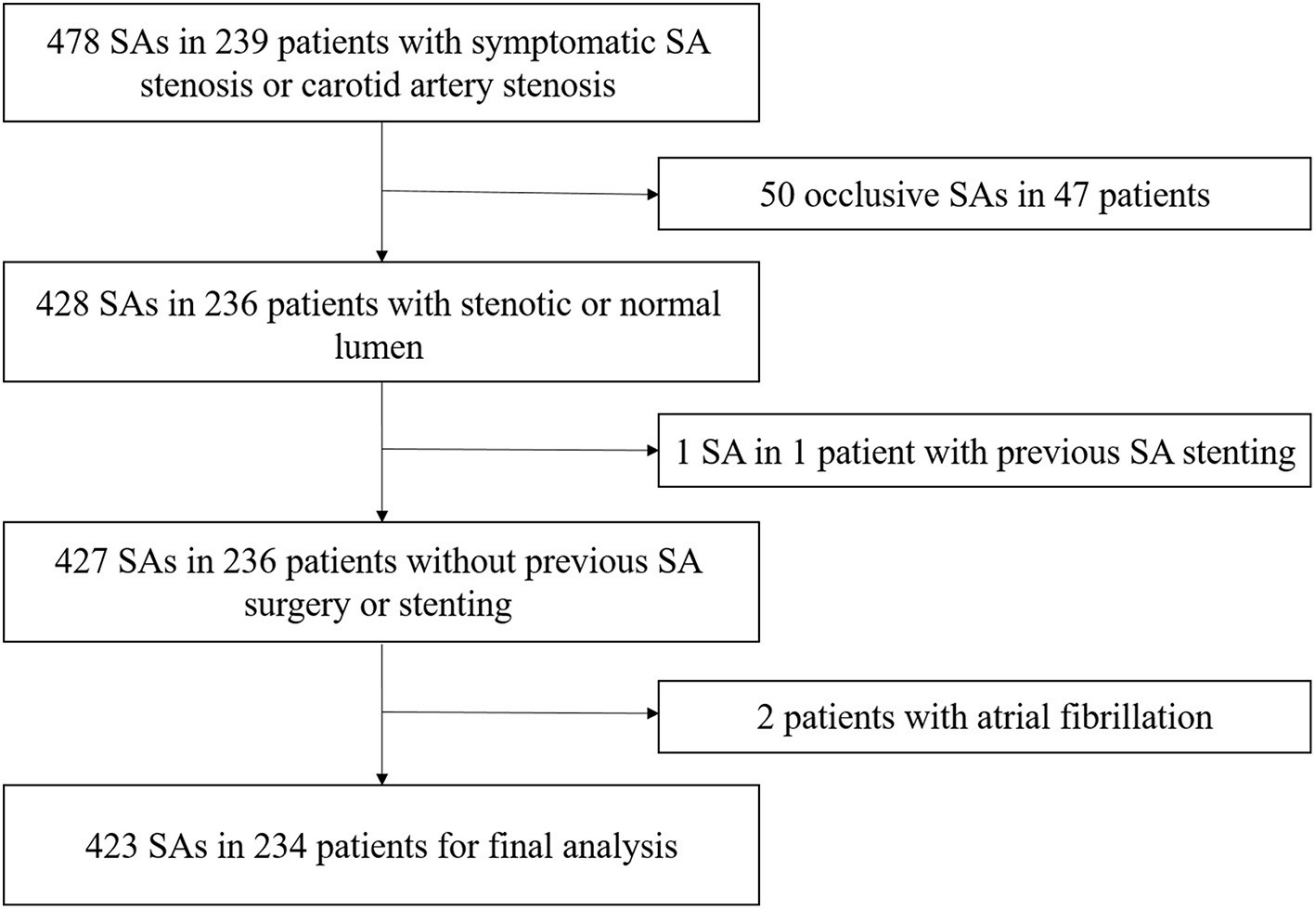
Study inclusion/exclusion criteria. SA, subclavian artery.

From April 2020 to August 2021, 239 consecutive patients with symptomatic SA stenosis or carotid artery stenosis who were scheduled to undergo DSA at our institution were prospectively enrolled (Figure 1). All the patients had undergone computed tomographic angiography (CTA) or magnetic resonance angiography (MRA) examination, which confirmed subclavian artery or carotid artery stenosis. The subsequent DSA was performed with a view to endovascular percutaneous transluminal angioplasty (PTA) or stent implantation in the same session. All patients were fully informed of the procedure and provided informed consent. They were evaluated by blinded examiners, first by CDU, and then by DSA, within an interval of 24 h. Data on patient demographics and vascular risk factors were collected after CDU. Symptomatic subclavian stenosis was defined as upper extremity claudication, vertebrobasilar insufficiency, myocardial ischemia in patients who underwent coronary artery bypass grafting utilizing the internal mammary artery, and lower extremity claudication in patients who underwent axillo-femoral bypass. The exclusion criteria were as follows: (i) SA occlusion, (ii) previous SA surgery or stenting, and (iii) atrial fibrillation or other arrhythmias affecting velocity assessment. The study protocol was approved by the ethics committee of the First Hospital of Jilin University on March 20, 2020 (approval number: 2020-632). The study was completed in compliance with the current privacy regulations.

### Ultrasonic Examination

**Figure 2:**
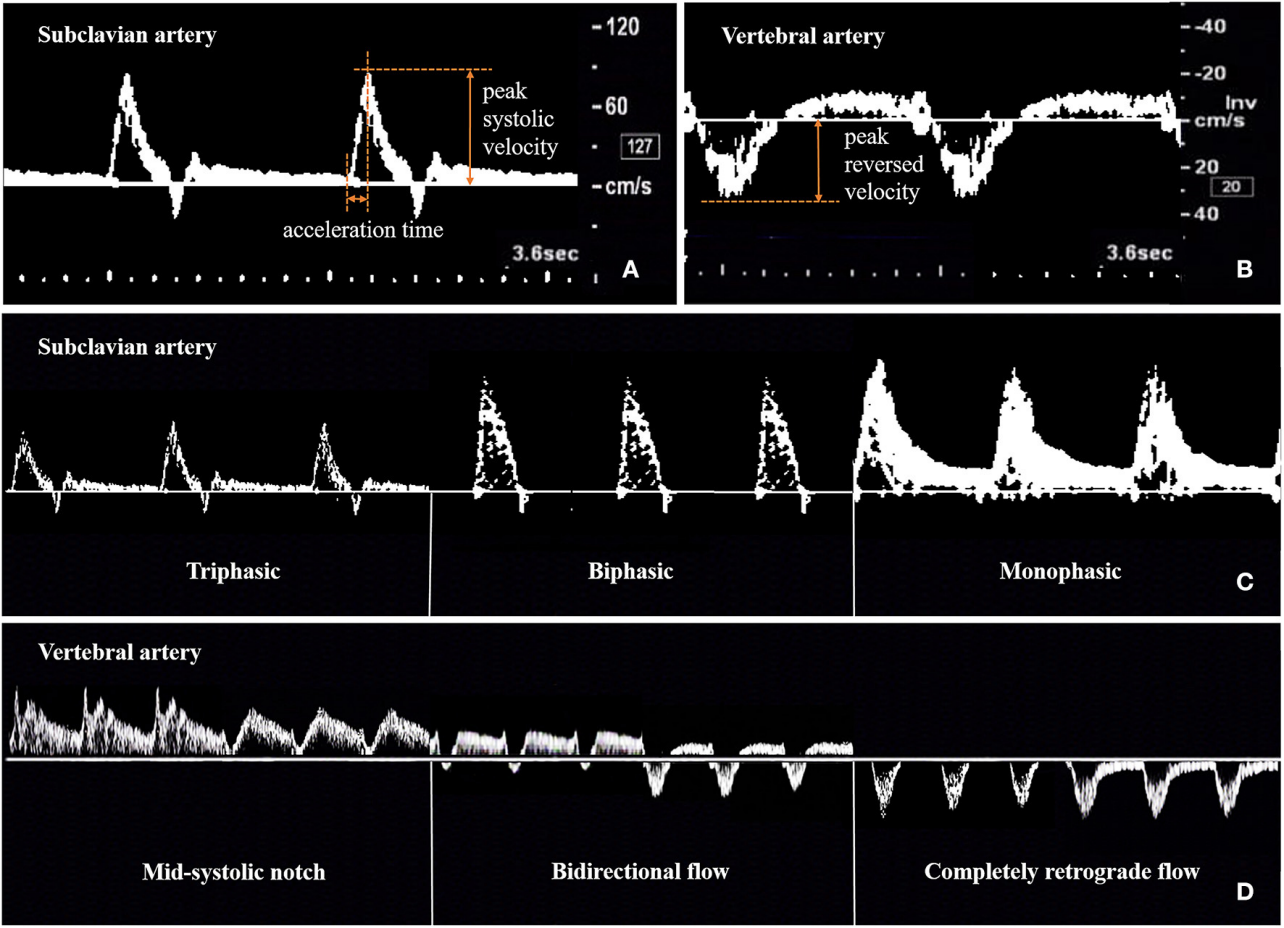
Method for measuring and recording ultrasonic parameters of subclavian artery **(A,C)** and vertebral artery **(B,D)**.

For CDU, an iU22 system (Philips Healthcare, Bothell, WA) with a 9-3 MHz linear array probe and 5-1 MHz curvilinear array probe was used. According to the ultrasound protocol previously described by Pellerito (21), all CDUs were performed by the same physician (Z. J.), who had 8 years of experience in vascular ultrasonography, had performed more than 2000 vascular cases per year, and who was unaware of the patient's clinical data (other imaging data, physical examination results, laboratory results, and patient history). The ultrasound parameters measured were peak systolic velocity (PSV) and acceleration time (AT) at the stenotic segment of the SA, peak systolic velocity (PSVd) and acceleration time (ATd) at the distal segment of the SA, and peak reversed velocity (PRV) of the VA. The spectral waveforms of the stenotic SA, distal SA, and VA were recorded. The ratios of PSV to PSVd (PSVr) and ATd to AT (ATr) were also calculated. AT represents the time from systolic acceleration to peak flow. PRV represents the value from the baseline to the reversed systolic peak of VA. The waveforms of the stenotic and distal SA included the triphasic, biphasic, and monophasic waveforms. The waveforms of the VA included the unchanged, mid-systolic notch, bidirectional, and completely retrograde waveforms (Figure 2).

### DSA Examination

DSA was performed using AXIOM Artis dBa (Siemens, Erlangen, Germany). All catheterizations were performed using a transfemoral approach with standard diagnostic catheters. After aortic arch injection, selective supra-aortic (carotid and subclavian) artery injections were performed. Angiography was interpreted by two experienced vascular physicians (C. Y. and W. S.), who had at least 5 years of experience in endovascular therapy. If the results were inconsistent, the final decision was made by another senior physician (W. L.), who had 12 years of endovascular therapy experience. All physicians were blinded to the ultrasound examination findings and other imaging data. The degree of SA stenosis was recorded, which was determined using the North American Symptomatic Carotid Endarterectomy Trial criteria (22). The degree of SA stenosis was subdivided into three categories: normal, 50–69%, and 70–99%.

### Statistical Analysis

Normality of distribution was assessed using the Kolmogorov–Smirnov test. Continuous variables are expressed as mean ± standard deviation or median (interquartile range), and categorical variables are expressed as frequencies and percentages. Categorical variables were tested using the χ^2^ or Fisher's exact tests, and continuous variables were tested using one-way analysis of variance or the Kruskal–Wallis test. The Spearman's rank correlation coefficient was used to calculate the relationship between the degree of SA stenosis and hemodynamic parameters. The predictive values (sensitivity and specificity) were calculated. The optimal thresholds of various hemodynamic parameters for SA stenosis were determined based on the maximum Youden's index by receiver operating characteristic curve analysis. Statistical comparisons of the area under the receiver operating characteristic curve (AUC) of the different hemodynamic parameters were performed according to Delong's test (23). Statistical significance was set at *P* < 0.05. The SPSS (version 26.0, IBM Corp, Armonk, NY) and MedCalc (version 19.5.6 for Windows, Mariakerke, Belgium) software were used to conduct the statistical analyses.

## Results

### Baseline Data

A total of 478 SAs in 239 patients were enrolled, including 78 patients with symptomatic SA stenosis and 161 patients with symptomatic carotid stenosis. Among the 156 SAs in 78 patients with symptomatic SA stenosis, 91 stenotic arteries and 65 contralateral normal arteries were included. Forty-three (55.1%) patients had concomitant carotid artery disease. Among the 322 SAs in 161 patients with symptomatic carotid stenosis, 57 asymptomatic stenotic SAs and 265 normal arteries were included. Overall, these patients included 148 stenotic SAs and 330 normal SAs. According to the exclusion criteria in Figure 1, a total of 98 stenotic SAs and 325 normal SAs were enrolled finally. No complications of DSA occurred. With combined use of the linear and curvilinear array probes, all the ultrasound parameters of enrolled patients were obtained. According to the results of the DSA, 50 (11.8%) had 50–69% stenosis, 48 (11.3%) had 70–99% stenosis, and 325 (76.8%) had normal lumen. Among the stenotic SAs, Eighty-nine (90.8%) SA stenosis were located at the origin, six (6.1%) were located near the origin of the VA, two (2.0%) were located distal to the VA, and one (1.0%) were multiple stenosis. Fifty-five (56.1%) SA stenosis coexisted with ipsilateral VA stenosis, two (2.0%) with ipsilateral VA hypoplasia, 28 (28.6%) with contralateral VA stenosis, 29 (29.6%) with contralateral SA stenosis, and one (1.0%) with an ipsilateral VA originating from the aortic arch. Fifty-four (55.1%) stenotic SAs were symptomatic, including 50 SAs with posterior cerebral insufficiency, two SAs with arm ischemia, and two SAs with coexistence of posterior cerebral insufficiency and arm ischemia. The proportion of symptomatic SA stenosis was the highest in the 70–99% stenosis group (60.4%), but this was not significantly different (*P* = 0.318) from the proportion in the 50–69% stenosis group (50.0%). Demographic characteristics are summarized in Table 1. There were no significant differences in the baseline data between the different groups (*P* > 0.05).

**Table 1:** Demographic characteristics.

	**Degree of stenosis**				
**Variable**	**Normal (*n* = 325)**	**50–69% (*n* = 50)**	**70–99% (*n* = 48)**	**F/χ^2^/H value**	***P*-value**
Age (years)	62.8 ± 9.3	62.9 ± 8.1	63.5 ± 7.5	0.124	0.883
Male	266 (81.8%)	38 (76.0%)	34 (70.8%)	3.698	0.157
**Comorbidity**					
Hypertension	210 (64.6%)	30 (60.0%)	23 (47.9%)	5.073	0.079
Diabetes mellitus	102 (31.4%)	16 (32.0%)	12 (25.0%)	0.844	0.656
Dyslipidemia	102 (31.4%)	20 (40.0%)	17 (35.4%)	1.618	0.445
Coronary artery disease	49 (15.1%)	5 (10.0%)	7 (14.6%)	0.906	0.636
Smoking	185 (56.9%)	28 (56.0%)	23 (47.9%)	1.376	0.502
Symptomatic	0 (0.00%)	25 (50.0%)^*^	29 (60.4%)^*^	207.675	<0.001

*Hypertension, blood pressure ≥ 140/90 mmHg; diabetes mellitus, fasting plasma glucose ≥ 7 mmol/L or 2 h postprandial plasma glucose ≥ 11 mmol/L; dyslipidemia, total cholesterol ≥ 6.22 mmol/L or triglycerides ≥ 2.26 mmol/L or low-density lipoprotein ≥ 4.14 mmol/L; smoking, smoked for >6 months; symptomatic SA stenosis includes posterior cerebral insufficiency and arm ischemia*.

* *Compared with the normal group, P < 0.05*.

### Hemodynamic Parameters

The features of SA stenosis detected by CDU were increased PSV, spectral broadening, and extended AT in the SA Doppler, a change from a triphasic waveform to a biphasic or monophasic waveform at the stenotic SA and distal segment, and mid-systolic notch or reverse flow at the VA. The hemodynamic characteristics are summarized in Table 2. A correlation was observed between the degree of SA stenosis and PSV (*r* = 0.624, *P* < 0.001), PSVd (*r* = −0.313, *P* < 0.001), PSVr (*r* = 0.654, *P* < 0.001), AT (*r* = 0.171, *P* < 0.001), ATd (*r* = 0.255, *P* < 0.001), and ATr (*r* = 0.128, *P* = 0.009). The waveforms (triphasic, biphasic, and monophasic) at the stenotic SA (*r* = −0.587, *P* < 0.001) and distal SA (*r* = −0.470, *P* < 0.001) were also related to the degree of SA stenosis. Forty-three SA stenosis (43/98, 43.9%) had concomitant ipsilateral VA spectrum changes. A correlation was found between the degree of SA stenosis and waveform of VA (*r* = 0.631, *P* < 0.001) and PRV (*r* = 0.483, *P* < 0.001). The presence of ipsilateral VA spectrum changes was significantly correlated with symptomatic subclavian stenosis (*P* < 0.001).

**Table 2:** Hemodynamic characteristics.

	**Degree of stenosis**				
**Variable**	**Normal (*n* = 325)**	**50–69% (*n* = 50)**	**70–99% (*n* = 48)**	**F/χ^2^/H value**	***P*-value**
PSV (cm/s)	147.0 (102.5)	350.5 (165.8)^*^	419.5 (196.8)^*^	164.233	<0.001
PSVr	1.3 (0.6)	3.0 (2.5)^*^	6.2 (4.1)^*^^,^^†^	180.983	<0.001
AT (ms)	50.0 (20.0)	50.0 (40.0)	70.0 (50.0)^*^^,^^†^	18.904	<0.001
ATd (ms)	50.0 (20.0)	50.0 (32.5)	105 (77.5)^*^^,^^†^	46.436	<0.001
ATr	1.0 (0.5)	1.0 (0.5)	1.4 (0.8)^*^^,^^†^	13.937	0.001
**Waveform of stenotic subclavian artery**					
Triphasic	284 (87.4%)	24 (48.0%)^*^	10 (20.8%)^*^^,^^†^	121.707	<0.001
Biphasic	41 (12.6%)	15 (30.0%)^*^	4 (8.3%)^†^	12.282	0.003
Monophasic	0 (0.0%)	11 (22.0%)^*^	34 (70.8%)^*^^,^^†^	228.433	<0.001
**Waveform of the distal subclavian artery**					
Triphasic	186 (57.2%)	14 (28.0%)^*^	5 (10.4%)^*^	46.204	<0.001
Biphasic	139 (42.8%)	28 (56.0%)	11 (22.9%)^*^^,^^†^	11.271	0.003
Monophasic	0 (0.0%)	8 (16.0%)^*^	32 (66.7%)^*^^,^^†^	155.970	<0.001
**Waveform of vertebral artery**					
Unchanged	323 (99.4%)	38 (76.0%)^*^	17 (35.4%)^*^^,^^†^	190.667	<0.001
Mid-systolic notch	2 (0.6%)	9 (18.0%)^*^	11 (22.9%)^*^	50.147	<0.001
Bidirectional	0 (0.0%)	3 (6.0%)^*^	10 (20.8%)^*^	42.618	<0.001
Completely retrograde	0 (0.0%)	0 (0.0%)	10 (20.8%)^*^^,^^†^	41.664	<0.001

*PSV, peak systolic velocity at the stenotic segment of subclavian artery; PSVr, the ratio of peak systolic velocity at the stenotic segment to that at the distal segment of subclavian artery; AT, acceleration time at the stenotic segment of subclavian artery; ATd, acceleration time at the distal segment of subclavian artery; ATr, the ratio of acceleration time at the distal segment to that at the stenotic segment of subclavian artery*.

* *Compared with the normal group, P < 0.05*.

† *Compared with the 50–69% stenosis group, P < 0.05*.

### Cutoff Values

**Figure 3:**
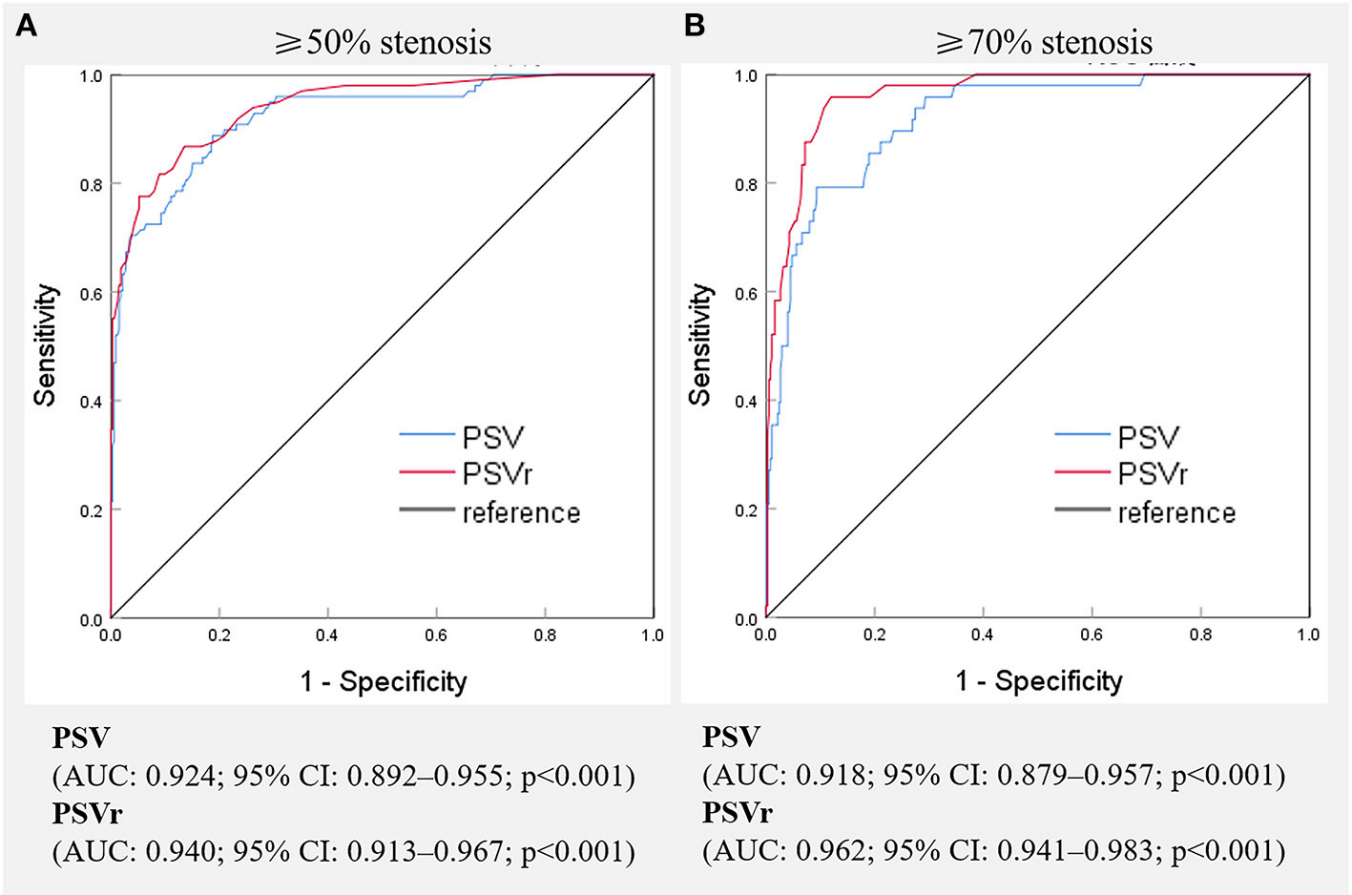
Receiver operating characteristic curves of various hemodynamic parameters for the evaluation of ≥50%**(A)** and ≥70%**(B)** stenosis of the subclavian artery. PSV, peak systolic velocity; PSVr, the ratio of peak systolic velocity at the stenotic segment to that at the distal segment; AUC, area under receiver operating characteristic curve, CI, confidence interval.

The receiver operating characteristic curves of SA flow for evaluating SA stenosis are shown in Figure 3. All AUCs were >0.90. For ≥50% stenosis, there was no significant difference between the AUCs of PSV and PSVr (*P* = 0.0872). The cutoff values were PSV ≥ 230 cm/s (sensitivity of 88.8 and specificity of 81.2%) and PSVr ≥ 2.2 (sensitivity of 86.7% and specificity of 86.5%). The sensitivity and specificity of changes of SA spectrum for predicting ≥50% stenosis were 65.3 and 87.4%, respectively. The sensitivity and specificity of changes of VA spectrum for predicting ≥50% stenosis were 43.9 and 99.4%, respectively. The parameters of SA flow were more sensitive than changes of SA or VA spectrum (all *P* < 0.001), but the change of VA spectrum was the most specific parameter (all *P* < 0.001). For ≥70% stenosis, the AUC of PSVr was larger than that of PSV (*P* = 0.0012). The cutoff values were PSV ≥ 340 cm/s (sensitivity of 79.2% and specificity of 90.7%) and PSVr ≥ 3.0 (sensitivity of 93.8% and specificity of 89.3%). The sensitivity and specificity of changes of SA spectrum for predicting ≥70% stenosis were 79.2 and 82.1%, respectively. The sensitivity and specificity of changes of VA spectrum for predicting ≥70% stenosis were 64.6 and 96.3%, respectively. The parameter of PSVr was the most sensitive parameter (all *P* < 0.05), and the parameter of change of VA spectrum was the most specific parameter (all *P* < 0.01).

## Discussion

In this study, we prospectively enrolled symptomatic patients with SA stenosis or carotid artery stenosis, and analyzed various ultrasonic parameters to predict SA stenosis. The results showed that CDU is an effective non-invasive method for evaluating SA stenosis. Furthermore, we established the grading ultrasonic diagnostic criteria for SA stenosis, with DSA as the reference.

### Ultrasound Parameters in Previous Studies

CDU is an appropriate evaluation method for patients with suspected subclavian occlusive disease. The ultrasound parameters for predicting SA stenosis include direct and indirect parameters. Most previous studies have focused on indirect parameters, including PRV and waveform changes of stenotic SA, distal SA, and VA. In the studies on changes of ipsilateral VA waveform, SA stenosis was predicted by evaluating retrograde VA flow (20, 24–28). Retrograde VA flow does not occur in all cases of SA stenosis. In our study, 43.9% of the SA stenosis had concomitant ipsilateral VA spectrum changes. Although the VA spectrum changes were related to the degree of SA stenosis (*r* = 0.631, *P* < 0.001), only 24.0% of the moderate SA stenosis and 64.6% of the severe SA stenosis were combined with VA spectrum changes. This was partly due to the high incidence of blocked VA-VA stealing pathway. The causes included ipsilateral VA stenosis (56.1%), contralateral VA stenosis (28.6%), contralateral SA stenosis (29.6%), SA stenosis located near or distal to the VA (8.2%), ipsilateral VA hypoplasia (2.0%), and ipsilateral VA originating from the aortic arch (1.0%). Although all the enrolled SAs were from patients who were scheduled to undergo DSA, which might cause selection bias, our study showed that it was not sufficiently sensitive to use the spectral change of VA to predict SA stenosis in patients who required further consideration for interventional therapy. In addition, changes in the VA spectrum do not always indicate a subclavian steal phenomenon (29, 30). The damped and monophasic changes at distal waveforms in the upper extremity are also a sign of significant SA stenosis, but this finding may be present in healthy patients with low-resistance arm circulation (31). In the current study, waveform changes of distal SA correlated with SA stenosis, but the correlation was not as strong as other parameters. Two previous studies evaluated the direct parameters to predict exceeding 70% stenosis of SA, including PSV and PSVr (18, 19). Their studies showed conflicting results. Hua et al. (19) evaluated 252 SAs and recommended a PSV of ≥343 cm/s and PSVr of ≥4.0 for evaluating ≥70% stenosis, while Mousa et al. (18) evaluated 245 SAs and found that a PSV of >240 cm/s had good sensitivity to detect >70% stenosis. The cutoff values of PSV differed greatly, which would worry the clinicians about the practical application of ultrasound parameters. More importantly, surgical or interventional treatment is indicated for symptomatic patients with ≥50% SA stenosis. Thus, the evaluation of ≥50% stenosis is more meaningful, but there is no relevant literature report. In addition, several studies found that AT was an useful ultrasound parameter to predict internal carotid artery stenosis, lower extremity peripheral artery stenosis, renal artery stenosis, hepatic artery stenosis and coronary artery stenosis (32–36). However, its efficacy in evaluating SA stenosis is unclear. In the current study, we found that the parameters of AT, ATd, and ATr were significantly correlated with SA stenosis, but the correlation was not as strong as other parameters.

### Ultrasound Parameters in Current Study

We evaluated the correlation between SA stenosis and all ultrasound parameters, including direct and indirect parameters. We found that all parameters were significantly correlated with the degree of SA stenosis, with PSV (*r* = 0.624, *P* < 0.001), PSVr (*r* = 0.654, *P* < 0.001), change of SA waveform (*r* = 0.587, *P* < 0.001), and change of VA waveform (*r* = 0.631, *P* < 0.001) having the strongest correlation. Furthermore, we determined the cutoff values of PSV and PSVr for detecting ≥50% and ≥70% SA stenosis, and calculated the sensitivity and specificity of parameters of VA and SA waveform changes. Our focus was about the cutoff value of ≥50% stenosis. The parameters of PSV and PSVr were the most sensitive parameters, and change of VA spectrum was the most specific parameter (all *P* < 0.001). Based on the maximum Youden's index, PSV and PSVr were more appropriate parameters to predict ≥50% SA stenosis. In addition, owing to the conflicting results of the two previous studies on ≥70% SA stenosis, we conducted the prospective study with a relatively large sample size to verify these results. In the present study, we examined 423 SAs and recommended a PSV of ≥340 cm/s and PSVr of ≥3.0 to detect >70% stenosis. PSVr was the most sensitive parameter (all *P* < 0.05), and change of VA spectrum was the most specific parameter (all *P* < 0.01). Based on the maximum Youden's index, PSVr was the most appropriate parameter to predict ≥70% SA stenosis.

### Clinical Significance

Revascularization is recommended in patients with symptomatic SA stenosis, so the identification of symptomatic SA stenosis is of great importance. As shown in Table 1, patients with ≥50% SA stenosis may be symptomatic, and there was no statistical difference in the proportion of symptomatic patients in the 50–69% and 70–99% SA stenosis groups. In our study, PSV and PSVr were more appropriate parameters to predict ≥50% SA stenosis. The cutoff values were PSV ≥ 230 cm/s and PSVr ≥ 2.2. Therefore, if the patient has symptoms such as upper extremity claudication, vertebrobasilar insufficiency, myocardial ischemia with CABG, and lower extremity claudication with axillo-femoral bypass, and the ultrasonic flow parameters of PSV ≥ 230 cm/s and PSVr ≥ 2.2, they should be considered for further verification by CTA or MRA, or by DSA with a view to PTA/stent implantation in the same session. In addition, even asymptomatic SA stenosis is associated with an increased risk of morbidity and mortality related to underlying atherosclerotic disease burden in other vascular beds (4–7). These patients should be treated aggressively with antiplatelet agents, high-dose statins, and antihypertensive agents (1). Consequently, SA stenosis, whether symptomatic or asymptomatic, mild or severe, should be closely monitored the progress of the lesion. In our study, we added hemodynamic assessment of ≥70% SA stenosis, which would be helpful for the clinical long-term management of patients with SA stenosis.

### Limitations

#### Our Study Has Several Limitations

First, hyperemia-ischemia cuff test was not performed in this study, which was a useful screening tool for the detection of subclavian stenosis causing VA steal syndrom. Due to the high prevalence of VA stenosis in patients enrolled in the study, we did not evaluate its utility in the current study. We will evaluate the efficacy of hyperemia-ischemia cuff test in evaluating SA stenosis in the further. Second, the patients with SA occlusion were excluded, which were part of patients with symptomatic SA disease. We excluded the patients with SA occlusion, since no flow seen on CDU at the occlusive segment of subclavian artery and several direct ultrasound parameters could not be detected. In the future, we will carry out the study of comprehensive ultrasound evaluation of SA occlusion to explore the clinical significance of ultrasound parameters of occlusive SA. Finally, all enrolled patients in this study were from the stroke unit of the Department of Neurology at the First Hospital of Jilin University. As this study was conducted in a single department at a single center, it may have caused selection bias. The patients in this study were mainly those with vertebrobasilar insufficiency and upper extremity claudication, while those with myocardial ischemia after CABG were not included. In the future, we will conduct a multi-center and multi-departmental prospective study to verify these results.

In conclusion, our study found that CDU is a reliable imaging modality for evaluating SA stenosis. Symptomatic patients with the ultrasonic parameters of PSV ≥ 230 cm/s and PSVr ≥ 2.2 need to be considered for further verification by CTA or MRA, or by DSA with a view to PTA/stent implantation in the same session.

#### DSA to Seek Revascularization

The recommended graded cutoff values can help in long-term management of patients with SA stenosis.

## Data Availability Statement

The raw data supporting the conclusions of this article will be made available by the authors, without undue reservation.

## Ethics Statement

The studies involving human participants were reviewed and approved by Ethics Committee of The First Hospital of Jilin University. The patients/participants provided their written informed consent to participate in this study.

## Author Contributions

JZ contributed to the study conception and design, data collection, analysis and interpretation, and drafting of the manuscript. YX and LC contributed to the study conception and design, analysis and interpretation of the data, and revision of the manuscript. SW, YC, and LW contributed to the data collection and revision of the manuscript. All authors contributed to the article and approved the submitted version.

## Conflict of Interest

The authors declare that the research was conducted in the absence of any commercial or financial relationships that could be construed as a potential conflict of interest.

## Publisher's Note

All claims expressed in this article are solely those of the authors and do not necessarily represent those of their affiliated organizations, or those of the publisher, the editors and the reviewers. Any product that may be evaluated in this article, or claim that may be made by its manufacturer, is not guaranteed or endorsed by the publisher.
